# Safe abortion services during the COVID -19 pandemic: a cross-sectional study from a tertiary center in Nepal

**DOI:** 10.12688/f1000research.50977.1

**Published:** 2021-02-15

**Authors:** Shreyashi Aryal, Samata Nepal, Sagun Ballav Pant

**Affiliations:** 1 Department of Obstetrics and Gynecology, Lumbini Medical College Teaching Hospital, Palpa, Nepal; 2 Department of Community Medicine, Lumbini Medical College Teaching Hospital, Palpa, Nepal; 3 Department of Psychiatry, Tribhuvan University Institute Of Medicine, Kathmandu, Nepal

**Keywords:** abortion, contraception, lockdown, pandemic, psychological distress, Nepal

## Abstract

**Background: **Abortion is an essential service, the need for which has increased during the coronavirus disease 2019 (COVID-19) pandemic. Because of the lockdowns at several periods, these services were hampered. This study analyzed the pattern of Safe Abortion Services (SAS) at a tertiary healthcare center during the first six months of the COVID-19 pandemic in Nepal.

**Methods:** This is a cross-sectional analytical study. We compared the pattern of safe abortion services between the first three months of the pandemic when a lockdown was implemented and the second three months when the lockdown was eased. Demographic and obstetric profile of women, their abortion choices, method of termination, difficulty in accessibility, and level of psychological distress were studied.

**Results:** A total of 52 women were provided SAS during the study period. The number of women coming for SAS during lockdown was 47.1% less than that after easing of the lockdown. During the lockdown, women came at a later period of gestation with a mean of 9.5 weeks compared to 7.5 weeks in the later three months. Because of fear of COVID-19, 19.2% (n=10) women opted for termination of pregnancy. Increased need of contraception was felt but 40% (n=12) had problems of accessibility. More women had probable serious mental illness during the lockdown period (p=0.008).

**Conclusion:** Lockdown during the pandemic decreased the number of women coming for SAS due to barriers in accessibility. Contraceptive needs are also increased but access is difficult. The need for safe abortion services and contraception has increased during the pandemic but the lockdown caused inaccessibility. Psychological distress is prevalent, and fear of COVID-19 has become a common reason for termination of pregnancy. This pandemic can be taken as an opportunity to provide and improve contraception and abortion accessibility, and quality with integration of mental health support.

## Introduction

Safe Abortion Services (SAS) in Nepal include pre and post counseling on abortion and contraception methods, termination of pregnancy, diagnosis, and treatment of existing reproductive tract infection, providing contraceptive methods as per informed choice, and then follow up for post abortion complication management ( National Safe Abortion Policy, 2003). SAS are essential healthcare services for women, but the COVID-19 pandemic caused all elective procedures to be suspended in hospitals which includes SAS as well. The Royal College of Obstetricians and Gynaecologists (RCOG) and International Federation of Gynecologists and Obstetricians (FIGO) guidelines suggest that abortion be kept under emergency services during this pandemic and be provided to all women in need ( Poon *et al.*, 2020; RCOG, 2020). During this crisis, the need for these services are higher because women are unable to make free reproductive health choices due to quarantine travel bans, lack of access to health facilities, fewer healthcare providers and loss of income. The need for contraception is higher as regular employment is interrupted and migrant workers have returned home in large numbers. There is additional risk of rape and domestic violence. In Nepal, the unintended pregnancy rate was 68 per 1,000 women of reproductive age in 2014 ( Puri *et al.*, 2016). If SAS are not available, these women may resort to unsafe abortion practices. Marie Stopes International (MSI) predicts 2.7 million unsafe Abortions in 2020 due to the closure of its services in 37 countries ( Cousins, 2020). This study was done with the aim of analyzing the pattern of SAS at a tertiary health center during the first six months of the COVID-19 pandemic in Nepal.

## Methods

### Study design

This is a cross-sectional analytical study done at Lumbini Medical College Teaching Hospital (LMCTH) which is a teaching hospital situated in hilly western Nepal and is a referral center for the surrounding districts like Gulmi, Syangja and Rupandehi.

### Participants

All women attending Gynecology outpatient clinic of LMCTH for first trimester abortion services during the first six months of the COVID-19 pandemic in Nepal were included in the study after informed consent.

### Ethical consideration

Ethical approval was taken from the Institutional Review Board of Lumbini Medical college (IRC-LMC 08-C/020) before the commencement of the study.

### Duration

Nepal reported its first case of COVID-19 in January 2020 and with an increasing number of cases, the government declared the first phase lockdown beginning from 24 March 2020. This lockdown was eased at different time periods after July 2020, as per the decision of the provincial government. In the first three months, a lockdown was implemented, and all non-essential services were shut down, including out-patient services in hospitals and essential services were open for limited hours only. After the first three months, the lockdown was eased allowing restricted use of non-essential services. Private vehicles were allowed to be used for essential services, but public transport was not operational. This study compared the pattern of SAS between the first three months of 2020 (April–June) and the successive three months (July-September).

The demographic and obstetric profile of these women about their abortion choices included the reason for termination of pregnancy and method of termination, difficulty they are facing relating to access to these services, and their level of psychological distress were studied.

### Study tools

A self-designed proforma was used for data collection and interview technique was used. Interviews were taken by the attending gynecologist who underwent an orientation program before data collection. The Kessler psychological distress tool, “K-6 tool”, which is a self-administered tool validated for Nepal, translated by experts in Nepali language ( Kessler *et al.*, 2003; Kessler *et al.*, 2010) [6,7]. This tool was used to find the level of psychological distress. The K-6 tool is a six-item self-report measure of psychological distress used to assess risk for serious mental illness in the general population answerable in five point scale. Using a 30-day reference period, respondents rated how often they felt nervous, hopeless, restless, or fidgety, so sad that nothing could cheer them up, that everything was an effort, and worthless.

The number of women coming for SAS three months prior to the beginning of the lockdown was analyzed using hospital medical records.

### Data analysis

Data was entered and analyzed using Statistical Package for Social Sciences (SPSS) v16. Results are presented in mean and SD, percentages. Relationship between categorical data was analyzed using chi square test. P value of <0.05 was considered as significant.

## Results

A total of 52 women were provided SAS during the study period. Three months prior to the lockdown (January–March 2020), 69 women were provided with SAS. Since the beginning of the lockdown, women coming for SAS were less by 25.7%.

Eighteen women came for SAS during the first three months and 34 cases came in the second three months. The number of women coming for SAS during lockdown was 47.1% less than that after easing of the lockdown.

The mean age of women was 26.1 years (SD=4.8), and the mean period of gestation was 8.23 (SD=1.98) completed weeks. Table 1 shows that more multigravidas came for SAS during both the periods. Employed women came for abortion more after the easing of lockdown (p=0.002).

**Table 1.** Demographic and obstetric profile of patients coming for SAS during the pandemic.

	During lockdown N=18 n (%)	After easing lockdown N= 34 N (%)	P value
Mean age (SD)	24.67 (4.08)	26.85 (5.15)	.476
Employed -Yes -No Job lost/breached due to COVID Yes No	5 (27.8) 13(72.2) 3 2	25 (73.5) 9 (26.5) 15 10	.002
Gravida -Primi -Multi -Grandmultipara (>5)	3 (16.7) 14 (77.8) 1(5.5)	7 (20.6) 26 (76.5) 1 (2.9)	.227
Previous abortion No Yes -Spontaneous -Induced	12 (66.7) 6 (33.3) 4 2	27 (79.4) 7 (11.8) 4 3	.334

During the lockdown, women came for abortion at a later period of gestation with a mean of 9.5 weeks compared to 7.5 weeks in the latter half of the study. 19.2% (n=10) women came for abortion because of fear of COVID-19. There was no significant difference in the method chosen for abortion (p=.378) in the two groups. Satisfaction towards services was found more after easing of the lockdown but this was not statistically significant (p=0.690) ( Table 2).

**Table 2.** Abortion characteristics during the pandemic.

	During lockdown n(%)	After easing lockdown n(%)	P value
Period of gestation	9.5 (1.72)	7.5(1.79)	.049
Reason for termination -Risk/Fear of COVID -Complete family -Education/Career -Poverty -Contraception failure -Out of wedlock -Sexual assault -Others	4(22.2) 10(55.6) 1 (5.6) 1(5.6) 1(5.6) 1(5.6) 1 (5.6) 0(00)	6(17.6) 13(38.2) 9(26.5) 3(8.8) 1(2.9) 1(2.9) 0(00) 1(2.9)	
Method chosen for termination: -Medical -Surgical	9 (50) 9 (50)	22 (64.7) 12 (35.3)	.378
Abortion outcome -Complete -Incomplete -Lost follow up	12 (66.7) 2(11.1) 4(22.2)	25 (73.5) 5(14.7) 4 (11.8)	.598
Complications -Present -Absent	2 (11.1) 16(88.9)	4 (11.8) 30 (88.2)	.661 *
Satisfaction of service -Satisfactory -Unsatisfactory	14 (77.8) 4 (22.2)	26 (82.4) 6 (17.6)	.690 *
Satisfaction towards service provider -Satisfactory -Unsatisfactory	10 (55.6) 8 (44.4)	27 (79.4) 7 (20.6)	.071

*Fisher exact.

Increased need of contraception was felt by 48.07% (n=25) women during the study period but there was no significant difference in the need of contraception during or after the lockdown. Out of 57.6% (n=30) women who did not use contraception, 40% (n=12) stated the reason of non-use to be inaccessibility due to lockdown. Injectable contraception was the most common family planning method chosen after abortion in both groups ( Table 3).

**Table 3.** Contraception use before during and after the lockdown period.

	During lockdown n(%)	After easing lockdown n(%)	P value
Contraceptive need increased Yes No	8 (44.5) 10 (55.6)	17 (50) 17 (50)	.703
Use in the past six months Yes No	5 (27.8) 13 (72.2)	17 (50) 17 (50)	.123
Reason for non use: Partner denial Fear of side effects Others. Non availability due to lockdown	3 (16.7) 4 (22.2) 2 (11.1) 4(22.2)	0 (0) 8 (23.5) 1 (2.9) 8 (23.5)	
Method chosen after abortion -Condom -Pills -Injectable -Implant -IUCD -Permanent -None	2 (11.1) 1 (5.6) 5 (27.8) 4 (22.2) 2 (11.1) 1 (5.6) 3 (16.7)	2 (5.9) 7 (20.6) 15 (44.1) 4(11.8) 4(11.8) 2 (5.9) 3 (8.8)	.077

Table 4 shows that women who lived more than five hours drive away from the hospital by public transport did not come for treatment during the lockdown period. 25% (n=13) women had to use an ambulance to reach the hospital. 21.1% (n=11) women had difficulty finding vehicles to reach the hospitals and 25% (n=13) women paid double the normal price to these vehicles. It was noted that transport costs double for some women (5 out of 16), even after the lockdown was eased. 17.3% (n=9) women had tried to get abortion pills from the local pharmacy before coming to the hospital.

**Table 4.** Accessibility to abortion facilities.

	During lockdown n(%)	After easing lockdown n(%)	P value
Distance of residence from hospital: -Within 2 hours drive -2-5 hours drive -More than 5 hours	11(61.1) 7 (39.9) 0	12 (35.3) 11 (32.4) 11 (32.4)	.021
Mode of transport to the hospital -Walking -Private vehicle -Public transport -Ambulance	11 (61.1) 3 (16.7) 0 4 (22.2)	9 (26.5) 9 (26.5) 7(20.6) 9 (26.5)	.585 *
Payment for transport (public/ ambulance) -Normal rate -Double rate -More than double	1 2 1	11 5 0	.585 *
Availability of transport (public/ ambulance) Easy Difficult	1 3	5 8	.622 *
Tried access to MA in pharmacy Yes No	4 (22.2) 14(77.8)	5(14.7) 29(85.3)	.495 *

*Fisher Exact.

**Table 5.** Psychological distress in women coming for SAS.

Psychological distress score (K6)	During lockdown n(%)	After easing lockdown n(%)	P value
Mean score (SD)	19.77 (6.81)	16.23(6.44)	.070 *
No probable serious mental illness (6-18)	12 (35.3)	14(77.8)	.008
Probable serious mental illness (19-30)	22 (64.7)	4 (22.2)	

*Independent sample t test.

The mean score K-6 score for all women coming for SAS during the study period was 17.4 (SD=6.7). The score was significantly more (p=.008) in women coming for SAS during the lockdown period. More women had probable serious mental illness during the lockdown period compared to those coming after the easing of lockdown (p=0.008) ( Table 5).

## Discussion

The COVID-19 pandemic has had a profound effect on sexual and reproductive health (SRH), and the findings of this study suggest that abortion and contraceptive services, which form a major component of SRH, have been affected to a great extent.

The findings of this study show that, because of travel bans, number of women coming for first trimester SAS was decreased by over 25% since the beginning of the lockdown. These women either have continued their pregnancy unwillingly or must have resorted to unsafe measures. The Guttmacher institute estimates that if 10% women who would normally come for SAS resorted to an unsafe method, an additional 3.3 million unsafe abortions would occur in low-middle income countries over the course of a year. This would in turn result in an additional 1,000 maternal deaths ( Riley *et al.*, 2020). With 25% women not coming for services in a referral center, as shown by this study, we can estimate that abortion related maternal deaths will increase.

Abortion was legalized in Nepal in 2002. It is available up to 12 weeks’ gestation on request, up to 18 weeks’ gestation in cases of rape or incest, and at any time if the pregnancy poses a danger to the woman’s life or physical or mental health or if there is a fetal abnormality ( National Safe Abortion Policy, 2003). Medical abortion was introduced in 2009 which increased access to abortion care. This was achieved after a long battle, and since then, Nepal has come a long way in providing SAS and reducing maternal mortality rate due to abortion complications. This pandemic seems to affect this hard-earned good outcome ( Wu *et al.*, 2017).

After easing of the lockdown, the number of women coming for SAS increased by just over 50% compared to the lockdown period. Women who came for abortion during the lockdown period had higher mean gestational age because they had problems of accessibility. Abortion is time sensitive as the higher the period of gestation, there are more chances of complications. They were not allowed to walk, had no means of transport to reach the hospital and, even if they hired a vehicle, they had to pay a higher price than normal even after the easing of lockdown.

Fear of COVID-19 is prevalent among people of all age groups. This study shows that 19.2% women wanted termination of pregnancy due to fear of COVID-19. There was no significant difference in women wanting termination during the lockdown or thereafter. Studies have shown that many pregnant women fear of COVID-19 and think it might affect their fetus ( Hossain *et al.*, 2020). This could be the main reason for women wanting termination during this time.

Some women chose termination because of financial reasons. Many women live under the poverty line in Nepal and more who did not hold an office job came for SAS in this study. Eighteen women were not working because of the pandemic and this was one of the reasons for pregnancy termination.

Incidence of gender-based violence has increased during this pandemic ( Raj *et al.*, 2020). During this study, there was one woman who came during the lockdown period who wanted termination of pregnancy occurring as a result of sexual assault. There were two women who wanted termination of pregnancy because of out of wedlock pregnancy. Both sexual assault and extra marital pregnancy are associated with social taboos in Nepal and can lead to increased psychological distress.

The findings of this study show that more women preferred medical abortion (MA) rather than surgical abortion during the pandemic, both during the lockdown and the period after that. The reason for this being less patient to service provider interaction and shorter hospital stays which was understood by both parties. Some women opted for surgical abortion because MA required a compulsory hospital follow up after two weeks. Abortion pills are easily available in local pharmacies in Nepal, but abortion has been de-medicalized legally for the purpose of easing accessibility to MA during the pandemic from May 2020. COVID-19 guideline for Maternal, Neonatal and Child Health (MNCH) has also been endorsed which allows home-based MA through outreach model and telemedicine. This will further increase the number of women choosing MA since pregnancy can be terminated without having to come to the hospital. This in turn will help mitigate accessibility problems and will be a relief for the over-exhausted health facilities concentrating mainly on the facilities concentrating mainly on the treatment of COVID-19 patients. Prescribing MA without an internal pelvic examination or an ultrasonogram would pose a risk of missing extra uterine pregnancies, so strict guidelines have been developed which needs to be strictly adhered to ( Raymond *et al.*, 2020).

More women who came for SAS during the post lockdown period were satisfied with the service and service provider when compared to the early lockdown period. Studies have shown that health care providers have moderate fear and burnout due to the pandemic ( Hu *et al.*, 2020), this could be the reason for the inability to provide satisfactory care to women coming for SAS. Especially during the initial days of the pandemic, when there was inadequate and low quality personal protective equipment (PPE), which was one major reason for inability of service providers to give satisfactory care.

Contraception is a major component of SAS, and the key area of concern is the increased need for contraception and its inaccessibility. Many women in this study felt an increased need for contraception and more so after the easing of the lockdown. One reason for this being the return of migrant workers from different countries and, at the same time, inaccessibility to contraception. Injection Depot Medroxyprogesterone was still the most popular choice among women coming for SAS which is the same as the national data of 2016 ( Pant *et al.*, 2019). Women did choose long-acting reversible contraceptives (LARC) as they could insert it in the same setting. Women have not been able to go for follow up visits and for refilling of their contraceptive pills. In this study, out of all women who did not use contraception, 40% had problems of inaccessibility.

Nepal is a hilly country with difficult terrain and lack of access for easy commutes, where some women have to walk for hours to days to reach a health facility even when they are not facing the pandemic. Accessibility to abortion and contraception becomes even difficult when there are travel bans. For women who are within reach, the closing of health facilities and pharmacies has posed a problem. Many women walked because transport was not easily available, and they had to pay more than the regular price. During the times of financial burden, this weighed them down more. The financial burden on the transport operators was passed down to the consumers and women coming to hospitals have to bear this brunt.

A study in pregnant women and their husbands showed that fear of COVID-19 was present in many couples which lead to depression and suicidal ideation in pregnant women. The findings of this study highlight that psychological distress was more during the lockdown period when compared to the time when lockdown was eased. Women having probable serious mental illness was high during the first three months ( Ahorsu *et al.*, 2020). During the time when the pandemic was announced, there was uncertainty about COVID-19, its pathogenesis, and complications. This caused increased mental pressure on many women, especially pregnant ones. As the situation was understood, the stress level decreased, and people began to normalize the situation. This could be the reason this study shows decreased levels of psychological distress in the later months. 

Increased psychological distress could have a serious effect on the long run on maternal mental health. Abortion itself is a traumatizing experience which has a devastating long-term effect on the woman and her family. Unsatisfactory SAS for a woman who has a chance of mental illness could be detrimental. Many women lost their follow-ups in this study; such loss of follow-up would compound the mental health effects of the pandemic and of SAS.

The limitation of this study is its small sample size so the significant results may have been due to chance alone. This study also does not address the issue of second trimester abortion. Due to inaccessibility, the need of second trimester abortions will be more than ever. A further analysis of second trimester SAS can be a scope for future studies so that the gestational limit for termination of pregnancy can be increased at least for the duration of the pandemic. Another issue to be analyzed in the coming months would be the use of MA through local pharmacies or through outreach means before concluding that women have resorted to unsafe measures by not coming to the health care center for SAS.

Women continue to become pregnant during this pandemic so guaranteeing resources to SAS is critical in preventing maternal deaths. It is a commendable effort by the government to recognize SRH services as essential and allow its operation but other areas must be strengthened like increasing supply chains, improving access, and using innovations like tele-health for medical abortion. Access of proper PPE and incentives may also help in tackling fear and exhaustion of service providers so that they can give satisfactory abortion services.

This pandemic can be taken as an opportunity to provide and improvise contraception and abortion accessibility and quality with an integration of mental health support. This can make SAS a dignified experience for women and their families.

## Conclusions

The lockdown during the pandemic has decreased the number of women coming for SAS due to barriers in accessibility. Contraceptive needs are increased but access is difficult. Medical abortion has been the more sought-after method for those who are undergoing SAS treatment. Psychological distress is prevalent, and fear of COVID-19 has become a common reason for the termination of pregnancy.

## Consent

Written informed consent for collection of data and publication of the participants’ details was obtained from the participants.

## Data availability

### Underlying data

Dryad: Safe abortion services during COVID pandemic in Nepal. https://doi.org/10.5061/dryad.3tx95x6fc ( Aryal *et al.*, 2021).

Data are available under the terms of the Creative Commons Zero "No rights reserved" data waiver (CC0 1.0 Public domain dedication).
