# Supplementary material for: Safe abortion services during the COVID -19 pandemic: a cross-sectional study from a tertiary center in Nepal
Source: F1000Res. 2021 Feb 15;10:112. [Version 1] doi: 10.12688/f1000research.50977.1 (PMC8042513; doi:10.12688/f1000research.50977.1)
Supplement: Peer review history

# Peer review: Reviewer response for version 1

**DOI:** 10.5256/f1000research.54079.r82476

**Published:** 2021-04-12

**Authors:** Evelyn Eisenstein

**Affiliations:** 1 University of the State of Rio de Janeiro, Rio de Janeiro, Brazil

Very interesting paper and again highlighting the importance of prevention and health education during adolescence, 

 The best time to change some of these sexual behaviours that all over the world was extrapolated during the Covid pandemics.

 The accessibility of free contraception is a MUST everywhere and should be part of public campaigns.

 SAS abortion in Nepal is a plus, here in Brazil abortion is still illegal due to religious and political reasons, notwithstanding women's rights.

 As the mean age was 26 y, they are not anymore young, but are they married (?) whose responsibility it is for the SAS decision? The couple? The husband? Free women's choice? 

 If I could just suggest adding in the discussion > the UN references 2014 > Operational Guidance for Comprehensive Sexuality Education and Chandra Mouli *et al. *20 years after the International Conf on Population and Development Where are we with adolescent sexual and reproductive health rights? Journal of Adolescent Health 56 (2015) S1-S6 > available at: http://dx.doi.org/10.1016/j.jadohealth.2014.09.015. ^1^

 It would be cheaper and safer to have contraceptives available and accessible and free (even during a pandemic) than safe abortions/ or even unsafe abortions and again divide the culprit between men and women, as they (men) also need sexual education, for sure.

Is the work clearly and accurately presented and does it cite the current literature?

Yes

If applicable, is the statistical analysis and its interpretation appropriate?

I cannot comment. A qualified statistician is required.

Are all the source data underlying the results available to ensure full reproducibility?

Yes

Is the study design appropriate and is the work technically sound?

Yes

Are the conclusions drawn adequately supported by the results?

Yes

Are sufficient details of methods and analysis provided to allow replication by others?

Yes

Reviewer Expertise:

Pediatrics and Adolescent Health and Medicine

I confirm that I have read this submission and believe that I have an appropriate level of expertise to confirm that it is of an acceptable scientific standard.

## References

[1] : Twenty years after International Conference on Population and Development: where are we with adolescent sexual and reproductive health and rights?. *J Adolesc Health*.2015;56(1 Suppl) : 10.1016/j.jadohealth.2014.09.015 S1-6 10.1016/j.jadohealth.2014.09.015 25528975

# Peer review: Reviewer response for version 1

**DOI:** 10.5256/f1000research.54079.r79657

**Published:** 2021-03-09

**Authors:** Suresh Kayastha

**Affiliations:** 1 Kathmandu Cancer Center, Bhaktapur, Nepal; 2 Nepal Korea Friendship Municipality Hospital, Madhyapur Thimi, Nepal

This article has addressed one of the important aspects of reproductive health i.e. safe abortion care in Nepal during the COVID-19 pandemic. As the pandemic and subsequent lockdown had significantly hampered the health care delivery process, safe abortion care service was also interrupted in Nepal. This article has tried to address change in the pattern of abortion care seeking behaviour, obstacles faced by clients during lockdowns and pattern of chance on abortion care services in two phases of lockdown. Though the sample size and study population is small, the results found are quite significant for the policymakers as government should make proper arrangement to deliver one of the essential health care services in Nepal

 This article is well written though some grammatical corrections and minor changes need to be made.

**1) ABSTRACT**

 The conclusion that improving safe abortion care services along with contraception and mental health support during the pandemic is quite true given the data and statistical analysis. It would have been better if the type of study (e.g. prospective or retrospective study) has been clearly mentioned in the methodology section. Some of the points in the conclusion section are a repetition of the findings in the results section (decreased the number of women coming for SAS, Contraceptive needs are also increased,  Psychological distress is prevalent, and fear of COVID-19 has become a common reason for termination of pregnancy.)

**2) INTRODUCTION**

 The link of the effect of the pandemic in Safe abortion care, contraception and the possible threat of unsafe abortion during the pandemic is quite impressive. It would have been better if the national guidelines from the government (if available) and national society (Nepal society of obstetricians and gynecologists -NESOG) regarding abortion care during pandemics has also been mentioned ( http://www.nesog.org.np/images/resources/nesog_guideline.pdf).

**3) METHODs**

*3a) Study design* – better to mention prospective/retrospective study.

*3b) Duration* – it would be better if we remove unnecessary paragraphs and better to focus on study duration only (no need to mention-the demographic and obstetric profile of these women about their abortion choices included the reason for termination of pregnancy and method of termination, the difficulty they are facing relating to access to these services, and their level of psychological distress were studied.)

*3c) Study tool* – some of the points made have no link with the study tool e.g. the number of women coming for SAS three months prior to the beginning of the lockdown was analyzed using hospital medical records.

**4) RESULT **

- The comparison made for women seeking abortion care before the commencement of lockdown and first 6 months of lockdown (69 vs 52) does not seem to be true. Point to be noted is that duration or time frame is different (3 months vs 6 months). Had we taken the same time frame, the result would have been different (see below at discussion portion)
- *Table 1- *Only mean age has been shown. It would have been more informative if age groups and numbers were also mentioned. Only limited demographic and obstetric profiles have been shown. For better result and interpretation, more parameters should be explored (e.g-marital status/ religion/ occupation /education status). These factors have a significant effect on abortion care in Nepal.
- Table 2- Only mean age has been shown. It would have been more informative if gestational age groups and numbers were also mentioned. Reason for termination- there is a single case in section “OTHERS”. It would have been more clear if that “other cause” was mentioned.

**5) DISCUSSION**

- The finding that number of women coming for first trimester SAS was decreased by over 25% since the beginning of the lockdown seems to be largely underestimated. There is difference in duration taken before (i.e. 3 months) and after ( i.e 6 months ) lockdown for this comparison . Provided that we compare 3 month before lockdown with initial 3 month of the lockdown, this difference would be 73.9% ( (69-18)/69x100).
- Paragraph 4, 6 and partly in paragraph 5, 7 are just description of what is seen in result. There is no need to restate the finding of result section at discussion portion. This is seen in almost all paragraphs.
- Need reference for –
  Paragraph 2- The Guttmacher institute estimates that if 10% women who would normally come for SAS resorted to an unsafe method, an additional 3.3 million unsafe abortions would occur in low-middle income countries over the course of a year.
  Paragraph 3- Medical abortion was introduced in 2009 which increased access to abortion care.
  Paragraph 4- Abortion is time sensitive as the higher the period of gestation, there are more chances of complications. 
  Paragraph 8-Abortion pills are easily available in local pharmacies in Nepal, but abortion has been de-medicalized legally for the purpose of easing accessibility to MA during the pandemic from May 2020. COVID-19 guideline for Maternal, Neonatal and Child Health (MNCH) has also been endorsed which allows home-based MA through outreach model and telemedicine.
  Paragraph 12-A study in pregnant women and their husbands showed that fear of COVID-19 was present in many couples which lead to depression and suicidal ideation in pregnant women

**6) CONCLUSION** – It is once again a repetition of the finding of the result section.

 I would like to thank the author to bring this topic in highlight as many women suffered during pandemics. This paper will definitely make policymakers aware of the problems faced by Nepalese women and make proper arrangements for good services during pandemics and lockdown in the future.

Is the work clearly and accurately presented and does it cite the current literature?

Yes

If applicable, is the statistical analysis and its interpretation appropriate?

Yes

Are all the source data underlying the results available to ensure full reproducibility?

Yes

Is the study design appropriate and is the work technically sound?

Yes

Are the conclusions drawn adequately supported by the results?

Partly

Are sufficient details of methods and analysis provided to allow replication by others?

Yes

Reviewer Expertise:

obstetrics and gynecology

I confirm that I have read this submission and believe that I have an appropriate level of expertise to confirm that it is of an acceptable scientific standard.
